# Understanding managerial behaviour during initial steps of a clinical information system adoption

**DOI:** 10.1186/1472-6947-11-42

**Published:** 2011-06-17

**Authors:** Charo Rodríguez, Marlei Pozzebon

**Affiliations:** 1 Area of Health Services and Policy Research, Department of Family Medicine, McGill University, 515-517 Pine Avenue West, Montreal, Quebec, H2W 1S4, Canada; 2 HEC Montréal, Department of International Business, 3000 chemin de la Côte-Sainte-Catherine, Montreal, Quebec, H3T 2A7, Canada

## Abstract

**Background:**

While the study of the information technology (IT) implementation process and its outcomes has received considerable attention, the examination of pre-adoption and pre-implementation stages of configurable IT uptake appear largely under-investigated. This paper explores managerial behaviour during the periods prior the effective implementation of a clinical information system (CIS) by two Canadian university multi-hospital centers.

**Methods:**

Adopting a structurationist theoretical stance and a case study research design, the processes by which CIS managers' patterns of discourse contribute to the configuration of the new technology in their respective organizational contexts were longitudinally examined over 33 months.

**Results:**

Although managers seemed to be aware of the risks and organizational impact of the adoption of a new clinical information system, their decisions and actions over the periods examined appeared rather to be driven by financial constraints and power struggles between different groups involved in the process. Furthermore, they largely emphasized technological aspects of the implementation, with organizational dimensions being put aside. In view of these results, the notion of 'rhetorical ambivalence' is proposed. Results are further discussed in relation to the significance of initial decisions and actions for the subsequent implementation phases of the technology being configured.

**Conclusions:**

Theoretical and empirically grounded, the paper contributes to the underdeveloped body of literature on information system pre-implementation processes by revealing the crucial role played by managers during the initial phases of a CIS adoption.

## Background

The purpose of this investigation was to examine the initial phases of a joint clinical information system (CIS) implementation in two Canadian university and multi-hospital health centres. The adoption of new information technologies (IT) currently constitutes one of the major institutional pressures on health care delivery systems. Health care organizations display information-intensive business processes wherein health professionals strive to keep abreast of the best possible medical knowledge [1]. At the same time, contemporary health care delivery involves an increasing degree of complexity, which becomes more and more difficult when using paper medical records [2]. In this context, the purchase and implementation of computerized information systems constitutes a major strategic decision, largely sustained in this sector by the assumption that IT should facilitate and improve the quality of healthcare delivery and overall organizational performance [3-5].

Being behind other industries with regard to IT diffusion [6], the degree of acquisition and use of IT in the health sector varies according to the type of technology and health care facility. In hospital settings, information systems still mainly address administrative purposes [7,8]. When developed, clinical IT solutions have been customized and discrete, "in-house" solutions that lead to fragmented patient information, often not readily accessible at the point of care. An intricate network of players within the IT market is thus currently behind the pervasive trend towards integration, encouraging people in the health sector to see highly complex packages such as clinical information system (CIS) as crucial to their business processes [9].

A CIS is presented as advanced configurable and integrated package software. *Configurable *refers to technologies that are highly parameterizable, that is they are built up from a range of components to meet the very specific requirements of a particular organization [10,11]. *Integrated *concerns the technology ability to put together and make accessible all the clinical information across the hospital. In other words, a CIS has been defined as the health care information system designed to incorporate and manage the clinical information pertinent to the delivery of patient care, including elements such as order entries, results reporting, care planning and clinical documentation [12].

Despite its potential benefits, successful CIS projects are scarce [13,14]. In fact, accounts about the failure of CIS implementations are quite common, and this regardless of the methodological care and/or high financial resources invested in their development [15-17]. As it has been pointed out, CIS adoptions are likely to fail when they are seen as "purely technical implementations" instead of complex social implementations, and/or when organizational and political issues have been neglected [18-22].

The study of the IT implementation process and its outcomes has received a lot of attention in the information system (IS) research field since the 1980s [23,24]. However, the examination of pre-adoption and pre-implementation stages of configurable IT uptake, i.e. the phases preceding the go-live and use of a new information system, and their possible repercussions to the success or failure of its whole implementation process, appear largely under-investigated [25]. For instance, Herold et al. [26] suggest that the perceptions and attitudes developed during the pre-implementation phase will strongly influence the other phases of the project, sometimes with huge negative consequences. Also, Pozzebon and Pinsonneault [27] draw attention to the critical influence that initial organizational decisions have on the trajectories built up by consultants and clients during a configurable package implementation. From a variance perspective, Abdinnour-Helm et al. [28] note in contrast that time with the firm and position, more than high levels of pre-implementation involvement, have a greater impact on attitudes towards an enterprise resource planning (ERP) implementation. Indeed, a better understanding of IT pre-implementation phases and its consequences for the chain of events that will follow the go-live appears to be necessary [29].

**Figure 1:**
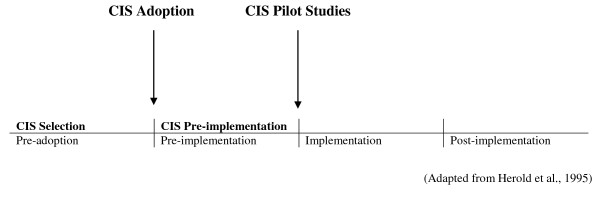
**CIS adoption and implementation timeline**.

This paper aims to bridge this gap, in particular concerning CIS projects. Accordingly, the question that guided the investigation was stated as follows: How do CIS stakeholders *initiate *and *set up *the new CIS? In other words, our objective was to identify the decisions and actions undertaken by top managers and other key CIS actors in order to select and prepare the effective implementation of a new CIS in two complex organizations, and to understand the intended and unintended consequences provoked by such decisions over two initial periods: CIS selection (or pre-adoption) and pre-implementation (see also Figure 1). We also examine the connections between the selection and pre-implementation phases, a subject virtually absent in the literature.

### A structurationist theoretical framework

Structuration theory has been extensively adopted by IS researchers over the last 20 years [30]. At the time of its formulation, Giddens' structuration theory [31] provided an account of the constitution of social life that challenged established theoretical positions and traditions [32]. Giddens departed from the conceptualization of structure as some given or external form. Structure is what gives form and shape to social life, but it is not itself the form and shape. Structure exists only in and through the activities of human agents [31]. Similarly, he departed from the idea of agency as something just "contained" within the individual; agency refers to the flow or pattern of people's actions. As Walsham notes [33], Giddens thus deeply reformulated the notions of structure and agency, emphasizing that "action, which has strongly routinized aspects, is both conditioned by existing cultural structures and also creates and recreates those structures through the enactment process". He suggested that while structural properties of societies and social systems are real, they have no physical existence. Instead, they depend upon regularities of social reproduction [34]. As a consequence, the basic domain of study in the social sciences consists of social practices ordered across space and time.

The same can be argued regarding the study of technological artefacts: technology is active only through human action. Accordingly, each CIS configuration decision is not merely technical, but social and political, affecting end-users' practices. In order to favour their acceptance, managers responsible for CIS implementations should therefore be conscious of the consequences of their technical choices on organizational practices. Furthermore, as noted previously, people (particularly physicians and other clinicians) who will be affected by such decisions should be listened to and truly involved in the entire process.

**Figure 2:**
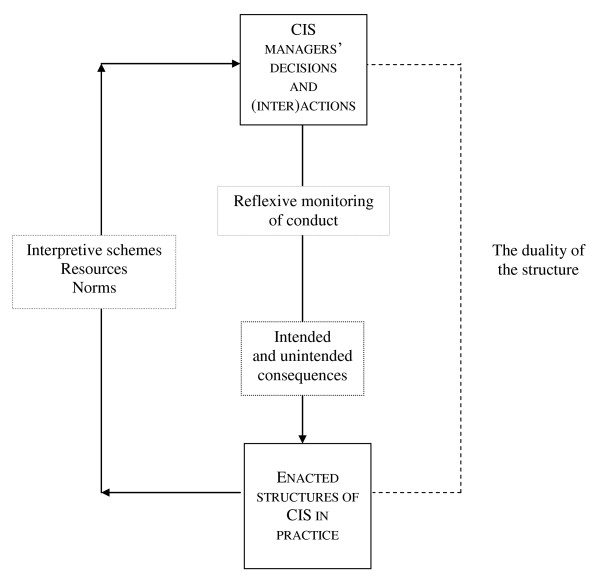
**A structurationist view of a CIS implementation**.

A structurationist framework can help understand and intervene in such contexts because it takes these concerns into account (see Figure 2). During the process of a new CIS configuration, knowledgeable CIS managers, able to reflect on their own and others' behaviour, will mobilize interpretive schemes, resources and norms in order to set up a collection of intentional and interdependent decisions and actions regarding future CIS operation. Each decision regarding the CIS configuration may have intended and unintended consequences, due to the fact that CIS managers, as human agents, always operate in a situation of bounded knowledgeability. The social nature of the CIS, or, in Orlikowski's terms [35], the enacted structures of the CIS-in-practice will thus progressively emerge from CIS stakeholders' decisions and (inter)actions, then recursively affect future CIS managers' decisions and actions concerning CIS adoption, and ultimately day-to-day clinical practices [36].

## Methods

Using Stake's typology [37], this is a collective case study, each multi-hospital system being a case. Considering that longitudinal research appears to be crucial for attaining a rich understanding of organizational change [38], this inquiry is better labelled a longitudinal collective case study. Appropriate ethical approval for the enquiry was obtained by the competent Institutional Review Board.

For the project phases under examination, i.e. CIS selection and pre-implementation, two complementary sources of data have been used: documents and archival material, and participant observations. The first method examines documents mainly comprised of the minutes of 41 meetings of CIS implementation committees that took place in the hospitals from the beginning of the CIS project in October 2001 to the end of the pre-implementation phase in June 2004 - 27 meetings of the CIS committee at the MHOSP1, and 14 meetings of the CIS committee at the MHOSP2. Moreover, archival material comprised of other organizational texts distributed before, during and after these meetings, such as CIS project management plan, CIS schemes of governance structure, working papers, and vendor's milestones were also examined.

The second method, participant observation, mainly concerns our regular attendance at these CIS implementation committee meetings at each hospital. Members of these committees were aware of who we were and the purpose of our investigation before accepting our presence at the meetings. Further, this has been a long-term full participant observation, in which we strike a balance in our role as pure observers with that of participants in the discussions [39]. The adoption of this method for more than two-and-a-half years has constituted a privileged opportunity for studying these groups of CIS managers in depth: it has allowed us access to a holistic view of the dynamic within their successive meetings, as well as of subjects' interactions within their particular committee settings. Indeed, our intensive diary notes have completed the body of texts considered in the study.

Two strategies have been used for analyzing textual data: discursive thematic analysis [40] and temporal bracketing strategy, the latest being considered a direct reference to Giddens' structuration theory [41]. Accordingly, we have first selected and described local pieces of texts and then we have interpreted them according to themes (or discursive "types") specific to the fields of IT and/or health services and policy - for instance, "end-users' involvement", or "CIS budgetary constraints". Drawing on other complementary texts from the context (e.g. governmental documents, newspapers), our prior knowledge of the institutional context within which these multi-hospitals evolved, and significantly, on our diary notes, we have elaborated a plausible explanation, trying to understand the power relations that underlie the production of those particular texts within their specific organizational and institutional contexts. Such systematic analysis across the corpus of texts allowed us to identify the decisions and actions that, over the period examined, have contributed to shaping the CIS to be implemented - see Table 1 for an illustration of this analytical procedure. Then, by bracketing this discursive activity over time, we have been able to examine how discourses have cumulatively contributed to the structuring of a new technological solution within an organization, i.e., how discourses in one period help to make sense of reality and lead to legitimated decisions and actions that influence the configuration being developed [27].

**Table 1:** Illustration of discursive thematic analytical procedure

Setting	Piece of text	Description	theme	Explanation
MHOSP1	*"[Dr. X] reiterated the need to have a Clinical Informatics group for the CIS, otherwise he feels that over time the CIS will lack in content if there is no continued input from clinicians."*	This physician is proposing and arguing in favour of the creation of a clinical informatics group in the hospital	CLINICAL MEANING OF THE CIS	This physician is very conscious of the importance of involving clinicians at this moment of the CIS project in order to give clinical meaning to the new system. He is advocating for the strength and legitimacy of local knowledge, which will support CIS acceptance in the hospital. However, although this proposal seems well accepted by certain members of the committee, CIS top managers have decided to postpone it to future CIS implementation phases.
MHOSP2	*"There are worries regarding the CIS adoption and the current management practices of access, the single sign-on, etc."*	Discussion about the impact of new CIS on current organizational practices	CIS IMPACT ON PRACTICES	This is a very animated discussion about the future impact of the CIS on current administrative and clinical practices on this hospital. Most of the members of the committee agree on the importance of this issue. However, discussions do not go beyond the highlighting of the importance of this issue.

## Results

### The CIS Project: Institutional Context and Project Stakeholders

MHOSP1 and MHOSP2 are the two biggest university multi-hospitals akin the Quebec public health care system. In response to their multiple missions --i.e. specialized care delivery, research, and teaching-- MHOSP1 and MHOSP2 have developed an impressive number of in-house and disconnected IT solutions and databases over the years. The need to acquire an integrated CIS had therefore been perceived in both university hospitals for years. However, the huge investment that such an acquisition represents had prevented its purchase. In such constraining circumstances, the decision to jointly adopt the same new CIS, made in fall 2001, was encouraged by the initial budget that a strategic research program, called here the "Rainbow Program", was ready to provide. Launched in July 2000 by the Canadian Foundation for Innovation (a federal research funding agency) over seven years, the Rainbow Program joins the efforts of research, clinical care and information services communities of several university hospitals in Quebec, in collaboration with various government agencies and private organizations. Its main goal is to create a clinical repository, a research data warehouse, and several research integration tools at the provincial level. The Rainbow Program - powerful in terms of material resources (a CDN $28 million program) and legitimacy within the provincial clinical and research community (it involves 15 clinical research groups and about 50 researchers) - requires an integrated information system for the entire health care network in order to develop its provincial data warehouse for clinical and health population research (its main objective). Indeed, the CIS project can be seen as part of the Rainbow Program. Nevertheless, due to the fact that the CIS is managed by MHOSP1 and MHOSP2 actors, the CIS project has always been presented by CIS top management as *their initiative*, whose main objective is the clinical management of patient data at an individual level, i.e. the adoption of an electronic medical record. In other words, the bargaining position of the CIS top management vis-à-vis the Rainbow Program is one of complementary partnership (i.e. interdependency), both being intertwined and needing each other in order to attain their respective goals: the former provides the data that the Rainbow Program needs for its provincial warehouse, and the latter brings material resources ($5 million per hospital) and increases the legitimacy of the CIS project.

Our involvement in the field for 33 months has enabled us to identify three main groups of CIS stakeholders (see Table 2). The first group is made up of the CIS top managers in each hospital, that is the Director of the CIS project, who holds an "umbrella" position over both multi-hospitals; CIS project managers from MHOSP1 and MHOSP2; and the chairs of the each institution's CIS committees, in operation since October 2001 and January 2003 respectively. The second group is composed of the members of each CIS committee: departmental managers from the different sites of each multi-hospital system, as well as clinical representatives (physicians and nurses) and managers from ancillary services, such as archives. Finally, the third group consists of the representatives of the Rainbow Program, who participate only in CIS committee's meetings at MHOSP1.

**Table 2:** CIS top managers

Groups	Description	How they are referred in the text
First group:	1. CIS Project Director	CIS Director
*CIS Project Top Managers*	2. CIS Project Managers (one from MHOSP1 and one from MHOSP2). They have the technical knowledge on the management of information systems projects	CIS Project Managers
	3. CIS Project Committees' Chairs in each MHOSP They have the organizational knowledge and legitimacy for coordinating the CIS project	CIS Committee Chairs

Second group: *CIS Project Committee Members*	4. Clinical representatives (e.g. physicians and nurses), departmental managers from the different sites of each multi-hospital system, and managers from ancillary services (e.g. archives)	CIS members

Third group: *External Actors*	5. Representatives of the Rainbow Program (only participating in MHOSP1 CIS Committee meetings)	Rainbow Program representatives

In the next sections, we present the description of the cases and our interpretations of how CIS managers, at each hospital, make sense of their organizational reality regarding the new CIS and how initial and ongoing decisions and actions influence the configuration being developed.

### MHOSP1 - The struggle between external pressures and organizational issues

The CIS project in MHOSP1 has been complex since the very beginning. In this hospital, CIS top managers' discursive practices, decisions and actions undertaken during the period prior to the CIS implementation suggest that, although aware of the impact and risks of the adoption of a new CIS on their organization, which will change flows of information and work practices, they have moved aside important organizational issues being raised by other CIS committee members, their decisions having been driven more by financial constraints and external interests. For a detailed depiction of these processes, we have broken down the first 33 months of the CIS journey in MHOSP1 into two major periods and five consecutive sub-periods.

#### The CIS Pre-adoption Period

##### October 2001-June 2002: Defining local requirements and interfacing with other hospital information systems

The selection phase of the CIS, led by CIS top managers from the MHOSP1 informatics department, began in October 2001. Two different methods were applied in order to favour a bottom-up process of identification of CIS requirements and facilitate a request for proposals according to clinical practices in use. First, an on-line 500-item questionnaire was mailed through their intranet to the more than 2,000 clinicians working in MHOSP1. The response rate was only 12%, with 93% of those reporting a positive attitude towards physician order entry (POE), and 76 clinicians volunteering to participate in focus groups (Meeting #3: January 18, 2002). In February 2002, 11 focus groups for detailing requirements were carried out. From the beginning, the low response rate to the questionnaire and the difficulties encountered in staffing focus groups echoed the difficulty of involving end-users in the CIS project. In any case, questions from the focus groups were compiled and organized for inclusion in the functional requirements document.

The complexity of interfacing the new CIS with existing information systems and the more than 300 databases in MHOSP1 necessitated an 'interfacing and transition team', which produced a document "that details the CIS interfacing requirements. This document gives a high level view of interfaces required prior, during and after various phases of the CIS" (Meeting #4: March 15, 2002). CIS top managers considered a *technical focus *emphasis of interfacing enough at this particular moment of the CIS adoption. Noting that "sites work differently sometimes", committee members' highlighted potential challenges (the emergency department) and alleged *organizational implications *to integration. CIS top management felt that "this was more an implementation issue than a request for proposals issue" (Meeting #5: April 19, 2002).

Another important issue discussed during the first sub-period of CIS adoption concerned the need for MHOSP1 to structure a permanent clinical information department to give *meaningful clinical content *to the CIS configuration. A physician, working in the hospital but attending CIS committee meetings as a researcher representative of the Rainbow Program, repeatedly raised this point.

"[Dr. X] shared his concern that at the MHOSP1 there is no Clinical Informatics group. He feels that this group should be a department (something that is permanent) not a committee. This group should be composed of multi-disciplinary and multi-professional users. This type of department would be required before we go live with a CIS. Many participants in the group were in agreement." (Meeting #3: January 18, 2002)

We consider that this issue is crucial in order to define the norms regulating the meaningful use of the CIS, and also to reinforce the local knowledge of CIS end-users vis-à-vis the global knowledge usually imposed by CIS vendors and external consultants. However, and despite the fact that most CIS committee members agreed upon this proposition, top managers cited funding constraints to postpone the issue to the implementation phase. As a result, the CIS was initially conceived as a purely technical device, the construction of its clinical meaning being postponed to subsequent phases of the CIS Project.

##### July 2002-March 2003: Selecting the vendor and worrying about funds

A Selection Task Force composed of 10 clinicians (physicians, nurses and allied health professionals) was created with the mandate of evaluating the 32 responses to the request for proposals according to four categories: technical infrastructure and interfaces, security, reports, and business proposal. Three of 32 met the minimum requirements (including technical as well as clinical, administrative, research and teaching requirements) and maximum cost allowance. Formal demonstrations took place in January 2003. The CIS vendor was selected by CIS top managers in March 2003, an event that marked the end of the pre-adoption phase.

Although the dominant issue during this sub-period was *the selection of the CIS vendor*, many concerns about *the financing of the CIS*, in terms of both the purchase of the system and the costs of its implementation became more and more explicit, raised in particular by nursing staff around the table:

"Many users have great concerns about the financing of the CIS. For the proper integration and implementation of a CIS, funds must be made readily available not only to purchase CIS software but also for process re-engineering, training, equipment (PCs, printers, etc.), extra resources, etc. The user community has yet to see a firm commitment from upper management of MHOSP1 indicating that funds will be made available for the proper implementation of the CIS." (Meeting #9: August 16, 2002)

Indeed, discourses regarding cost issues also dominated this second sub-period of CIS pre-adoption. CIS is a very costly IT, so top managers selected their CIS with the best balance between minimum organizational requirements and maximum cost allowance in view. Nevertheless, a CIS implementation cost involves not only the cost of the technology itself, but also the cost of training and organizational change that its adoption implies. Focusing on technological aspects of CIS adoption, the discussion about these organizational costs was, again, postponed by top CIS managers to following phases of the project.

#### The CIS Pre-implementation Period

##### April-October 2003: Striving for funds and working on the CIS project plan

Once the CIS selection (or pre-adoption) phase was finished, an unexpectedly long pre-implementation stage began. Contract negotiations began and the definition of the project scope was undertaken at each MHOSP. These processes were heavily tainted by the struggle to obtain a clear budgetary compromise from hospital upper management for the CIS once it was already selected.

The battle for financial *resources *for the CIS led to the elaboration by CIS top managers of three different budgetary scenarios: Plan A, advance funding from the budget for a new MHOSP1 site; Plan B, a 10-year loan authorization; and Plan C, funds from the hospital foundation (Meeting #20: September 2003). At the same time, a global budget increase from CDN $15 M to $17 M per site was accepted by hospital upper management, and funding from the Rainbow Program of $3.4 M in the first year of implementation remained secured (Meeting # 21: October 10, 2003). No clear decision regarding the source of the bulk of the CIS budget was made at that time. These were the circumstances that progressively placed the CIS stakeholders in a very difficult position regarding the *resources *needed for successful CIS adoption. Concerning the scope of the CIS implementation project, three major phases were defined: Phase 1 included 'results reporting' and 'data warehouse'; Phase 2 consisted of piloting the project in 1 or 2 clinical programs for 'order entry' and 'clinical notes'; finally, Phase 3 concerned rapid 'order entry deployment' and a more gradual 'clinical notes deployment' (Meeting #17: May 16, 2003). A few weeks later, clinical documentation deployment was segregated to a fourth Phase (Meeting #18: June 6, 2003).

From our examination of this process, it appears that CIS managers did not adequately connect the selection and pre-implementation phases: there appeared to be no plan to use the information gathered from the users to legitimate the selection in order to facilitate the fit with the system's functionalities. Clinicians' strong resistance to the introduction of a new CIS is well documented in the literature and is cited as one of the strongest contributors to CIS failure. In order to deal with this, strategies for *end-users' involvement *are strongly advocated [23,24]. In MHOSP1, this issue is often mentioned by CIS committee members, who repeatedly seek economic incentives to support physicians' involvement in the project and request careful analysis and discussion of the changes that will impact physicians' and nurses' practices over the different phases of the CIS implementation. However, always alleging budgetary constraints, CIS top managers put off this discussion about physicians' involvement until the implementation phase.

Indeed, the analysis of CIS top managers' discursive practices suggests they were much more concerned with the expected outcomes of the CIS than with the process of its adoption. Furthermore, they seem to lead the issue of *end-users' involvement *in a purely instrumental way: focusing on user involvement is important to increase the chances of project success and then to satisfy *the needs of Rainbow Program members*, as appears illustrated by the following excerpt:

"The CFI [Canadian Foundation for Innovation] is providing funds to the [Rainbow Program]. However, it is a high-risk project and the CFI will benchmark its evolution, as well as the CIS Project in that regard. Since point-of-care data collection is necessary for the [Rainbow Program], the CIS needs to feed research with this detailed data. This is the essence of the [Rainbow Program]. Hence, for clinical notes, a specialized [Rainbow Program] front-end has to be integrated into the CIS. This needs to be part of the initial roll out (Phase 1) if we do not know to lose the funding from CFI." [Meeting #17: May 16, 2003]

##### November 2003-January 2004: Negotiating the contract with the vendor

Contractual agreements with the vendor were established under the pressure of budgetary constraints. In this context, questions relative to timeline and the scope of services to be provided by the vendor were discussed. The issue of the intellectual property of the system also attracted great attention:

"[CIS top manager] indicated that for the changes for research, the idea will belong to the researcher but the incarnation of these ideas into the [CIS] product will then belong to [the vendor]. [CIS top manager] indicated that lawyers have looked into this issue and that for [the vendor] this is a showstopper. They will not sign a contract if it means that MHOSP1 and MHOSP2 will get royalties from the sales of their [CIS] product to other customers." [Meeting #22; November 14, 2003])

In addition, the vendor privileged a "vanilla" CIS implementation mode, meaning that the software is taken *as is*, and a maximum of 30% customization would be tolerated. In this situation, the fit with the software will be achieved *by changing user practices*. Making such changes is always risky and complex, particularly when it involves *harmonizing *local practices with those of a quite different organizational culture [42]. These initial management decisions create a scenario in which careful rethinking of business practices and changes in management will be necessary in order to achieve standardization, not only internally, but also across the different hospital sites and institutions. Our fieldwork also suggests that, although CIS committee members repeatedly raised the issue of the need to compile a list of business processes to be "re-engineered" from the early moments of the selection phase, a careful discussion of how and when this would occur was always postponed by CIS top management.

At the end of this sub-period, and again mainly due to budgetary constraints, the envisioned structural properties of the CIS to be implemented included a contractual imperative of at least 70% CIS standardization in both hospitals (outcomes). Discussion on the processes through which the respective organizations had to reconsider their current business practices, however, was still relegated to future phases of the project.

##### February-June 2004: Critical power struggle between external and internal CIS stakeholders

What significantly increases the complexity of the CIS project in the MHOSP1 setting is the presence of an explicit "battle" between internal and external actors: between CIS managers and Rainbow Program representatives. The former sought *autonomy *vis-à-vis the latter, and emphasized the *meaning *of the CIS for the hospital at the individual clinical level (i.e. electronic medical record). For the latter, the CIS *had no individual meaning*, but just meaning as a tool for *aggregating data at clinical and population levels *(i.e. as data repository). The battle of interests, present from the beginning of the CIS project, intensified during the sub-period prior to signing the contract with the vendor. In this sense, and due to the lack of budget for the whole CIS project implementation, a 'reduced scope' was envisaged, favoured mainly by external CIS stakeholders:

"The funding of [the Rainbow Program] ends in March 2006 and the [Rainbow Program] must have something to show, such as data flow, research data study, etc. With this in mind, we could sign a reduced scope contract with [the vendor] so that we get an early start. The reduced scope would be a scaling down of the project for research only. This scaled down scope still needs to be worked out so as to clearly identify how we would proceed with the deployment to the wards and the clinics for the [Rainbow Program] only." (Meeting # 23: February 23, 2004)

A new phase, called Phase 1A, was then established and added to the contract with the vendor. However:

"It is indicated that the lengthy negotiations between [the Rainbow Program] and MHOSP1/MHOSP2 was becoming a problem for [the vendor]. [...] There is more and more pressure to sign a reduced scope contract between [the vendor] and the 2 MHOSPs whether the [Rainbow Program] contract is signed or not. [...] [The Rainbow Program's representative] indicated that [the Rainbow Program] wants to participate directly in all discussions between [the vendor] and the 2 MHOSPs if things are to move forward faster." (Meeting #26: May 14, 2004)

As a result, MHOSP1 was placed in a clear *dependent position *vis-à-vis the Rainbow Program (i.e. external CIS stakeholders) during the weeks before signing the contract with the vendor. MHOSP1 depended, on the one hand, on the Rainbow Program for funds and on the other, on the CIS vendor for *global *CIS *knowledge*. Furthermore, this dependency on external CIS stakeholders had to be added to the lack of internal mobilization of CIS end-users. Indeed, the signing of the contract between both MHOSPs and the CIS vendor took place in June 2004, *before *the signing of the contract with the Rainbow Program in July 2004, thanks to a bank loan that would allow CIS hospital top managers to undertake the implementation of Phase 1A, while waiting for funds for the whole CIS project.

### MHOSP2 - The "easy-going" battle between IT analysts and business managers

Compared to the power struggles at MHOSP1, quite a different scenario was found at MHOSP2. First, the CIS committee in this hospital was constituted just a few weeks before the CIS selection. Second, the absence of external actors --the Rainbow Program representatives participated only in MHOSP1 meetings-- attenuated the power struggles, because here there were fewer conflicting interests. In MHOSP2, we have broken down the period examined into three bracketed sub-periods.

#### The CIS Pre-adoption Period

##### January-March 2003: The CIS selection process

The discussions held during the first meetings of the CIS committee in MHOSP2 mainly involved monitoring the CIS selection ("The process of CIS selection is still going on, so the final decision is not made yet." [Meeting #2a: February 26, 2003]). Along with this, members of this committee expressed, from the beginning, their concern about *the impact of the CIS adoption on their business processes*, as illustrated in the following excerpt:

"There are worries regarding the CIS adoption and the current management practices of access, the single sign-on, etc. A discussion is held around current work on a number of applications. The preferred strategy is the following: the CIS utilization to integrate clinical information rather than the implementation of a single sign-on technology for a set of applications." (Meeting #2a: February 26, 2003)

In sum, the pre-adoption phase of the CIS was shorter and less complex in this hospital than in MHOSP2. Indeed, most of the work with regard to the CIS selection was made by the CIS top managers of this hospital in collaboration with their counterparts in MHOSP1, prior the creation of the MHOSP2 CIS planning committee.

#### The CIS Pre-implementation Period

##### April 2003-January 2004: Negotiating with the vendor and analyzing work practices

Once the CIS was selected, MHOSP2 actively participated in the process of negotiation with the vendor. In this sense, CIS budgetary uncertainty and delays due to changes at the political government level were highlighted as puzzling:

"Negotiations advance slowly. Certain crucial points remain unclear, in particular the date when the French version of the system will be available. In addition, the members of the committee are worried regarding funds. This appears very problematic, particularly within the context of the current budgetary exercise at MHOSP2 and the political context tied to the change in provincial government." (Meeting #7a: May 28, 2003)

During the CIS committee meetings at MHOSP2, it appeared clear that, although external legitimacy and image were important aspects of the CIS project, CIS top managers were much more concerned with their *internal needs*. Their stated purpose was to implement a CIS that will provide accurate clinical information and help improve clinical activities. Despite such concerns, a critical element emerging from the project was the need to harmonize the CIS package to at least 70% of the other sites. The organizational and political impact to do so appeared to be, at this moment, underestimated. For instance, CIS committee members' discursive practices during this sub-period showed a high concern with the possible effects of such high standardization on users' day-to-day practices and on how end-users might react to these changes. Nevertheless, no clear plan to deal with these organizational issues at an operational level was discussed. Still, such concerns sometimes appeared to be trivialized:

"[The vendor] agrees to an important reduction, which brings them to the same monetary level of their initial offer, although the scope of the project is now much more important than that included in the proposition. One of the conditions of this reduction is that 70% of the system must be identical for MHOSP2 and MHOSP1. [The CIS project director] indicates that the hospital CEO does not see any inconvenience to this constraint." (Meeting #9a: September 24, 2003).

##### February-June 2004: The battle for funds and the search for physician involvement

As noted previously, the issue of end-user involvement, and particularly that of physicians, was constantly discussed in the MHOSP2 meetings, especially once the decision to undertake Phase 1A was made. The committee decided to focus on physicians and other health professionals, members of the clinical unit pilots, in the reduced-scope CIS implementation. Top managers were also very concerned with strategies of *communication*, apparently aware that distorted messages can be very detrimental to the future acceptance of the CIS.

In addition, the battle for funds continued. Although the Rainbow Program representatives never attended MHOSP2 CIS committee meetings, these negotiations had a great impact on committee discussions. Indeed, when support from upper management was obtained for funds in Phase 1A --in other words, when they reached a greater bargaining power-- MHOSP2 favoured signing the contract despite unresolved misunderstandings with the Rainbow Program:

"MHOSP2 has already obtained the support from its Board of Governors in order to request the budget necessary for Phase 1A, and is seriously considering signing the contract with [the vendor] without waiting for the conclusions of the negotiations with [the Rainbow Program]. It is not clear at this moment if MHOSP1 will be able to do so. A last chance meeting will take place with [the Rainbow Program] on Friday, May 29." (Meeting #14a: May 26, 2004)

## Discussion

In contrast to functionalist social theory, which dilutes human agency under the deterministic effect of social structure, Giddens' structuration theory explicitly enhances social actors' intentionality, knowledgeability and reflexivity: human agents have reasons for acting as they do, although such purposeful behaviour involves unintended consequences regarding the recursively-related social structure, which in turn will affect future conduct. As Giddens notes, it is the examination of "the follow of intentional conduct" that allows us to foresee and explain possible future "unintended consequences" of our actions [31].

When applying these ideas in the present project, we can therefore state that CIS top managers' flow of decisions and actions during the CIS selection and pre-implementation phases have over time led to (1) financial dependency vis-à-vis external stakeholders, (2) a focus on technical issues and (3) a disregard (despite awareness) of organizational issues of the CIS adoption. All these have been *conscious and intended decisions and actions*, made by virtue of their power position and particular interests in the project, and largely justified by them in the present circumstances, i.e. institutional IT imperative and severe financial uncertainty. In doing so, CIS top managers not only have intentionally shaped the initial phases of the CIS implementation but also, according to a structurationist stance, set a frame within which future phases of the CIS implementation can be reasonably foreseen to be particularly complex.

In view of the high financial uncertainty that has surrounded the initial phases of this CIS Project, its top managers have been able *to avoid making decisions *on end-user involvement by recurrently postponing the discussion on this issue to subsequent phases of the CIS project (MHOSP1), or undertaking no concrete action to put user concerns into operation (MHOSP2). To do so, they have mobilized their *nondecision-making power *to exclude from the discussion such threatening issue in the current situation, without the risk exposure of making a formal decision in this regard [43]. In effect, most strategies to get physicians involved in CIS implementations require a non-negligible financial envelope for paying overtime so physicians can dedicate time to the project, or hiring new clinical staff with highly developed computer skills. Under financial uncertainty, CIS top managers have thus convinced themselves and the other participants that this issue could be addressed in further phases of the project. Yet the discursive activity they have shown has protected them from censure for their ongoing decisions, even if ultimately they were conscious of their potential negative consequences on following phases of CIS adoption.

Also, avowing the importance of the organizational side of CIS projects, CIS top managers' decisions and actions have shown a *clear emphasis on technical aspects of the CIS implementation*. This has mainly been, again, because the dominant issue of budgetary constraints and the dependency on external actors were out of their control and could potentially obstruct the accomplishment of a project that is highly relevant for them. In this context, we suggest that the *separation *between technical and social issues may constitute a discursive strategy for concealing problems and barriers that could impede the continuity of the project during pre-implementation phases.

Operating in a very uncertain context, and positioned in a clear external dependency regarding financial resources, these CIS top managers have nonetheless displayed the remarkable ability to create an internal organizational space for decision and non-decision making with regard to the CIS project over time. We call this managerial discursive ability *rhetorical ambivalence*. Rhetoric has lately attracted much attention among organizational scholars [44]. According to this body of literature, rhetorical strategies are inherent to any managerial activity [45]. This assertion would be particularly pertinent in knowledge-intensive organizations --as hospitals are-- due to the intense *ambiguity *that characterizes these organizational settings whose actors must struggle with [46]. We therefore argue that rhetorical ambivalence is one of the rhetorical strategies that managers would display in particularly ambiguous and uncertain organizational contexts. As it has been in the present empirical case, the CIS top managers, mastering and exhibiting rhetorical ambivalence over the CIS pre-implementation phases examined, have not only been able to convince of the imperative for their organizations to acquire a new CIS but also to select and negotiate the contract with the CIS vendor without budget availability, and commit both organizations to a long period without guarantee that funds for the project would be released. Furthermore, they have done so while preserving/restructuring their powerful position in the project.

It is important to note here that, with regard to financial resources, rhetorical ambivalence has proven to be successful in the mid-term as the budget for the project was finally approved by governmental authorities in fall 2009. At that time, Phase 1 of CIS implementation, i.e. visualization of results, was almost completed in both hospitals, with an increasing but still partial uptake among clinicians. However, rhetorical ambivalence *might *not be without negative consequences in the following phases of the CIS Project --the CIS master implementation plan includes four consecutive phases, hospitals having initiated or being to the point of initiating Phase 2 (i.e. computerized physician order entry) at the time of writing these words. From a structurationist perspective, we consider that although we can always change the chain of events with new decisions, actions and interventions --a possibility that is foreseeable here due to very slow pace of this CIS implementation-- these changes are within a given range; they have limits. Accordingly, the adjustment between technology and clinical practices, as previously mentioned, appear**s **to be particularly difficult in this project --the CIS uptake by end-users is rather modest to date, and it appears to be particularly scarce among nurses.

Although the emphasis on technical issues is rather frequent in similar projects [47], it is also well known that CIS implementations are likely to fail when organizational and political issues are neglected --in this sense, propositions from certain CIS project members to create new formal structures within the hospital to work with the vendor in order to configure a CIS meaningful at a clinical level have been avoided by CIS top managers during the periods examined. Moreover, this technical emphasis is being built around two ideas of the main goal of the CIS that correspond to the interest of two different, although not necessarily incommensurable, groups of CIS actors: a CIS as a means for aggregating data at clinical and population levels (the ultimate goal of the Rainbow Program), and a CIS as a tool for improving medical practices (the ultimate goal of MHOSP1 and MHOSP2). Such different visions may generate difficulties in future phases of the CIS implementation.

## Conclusions

Although the study of the IT implementation process and its outcomes has received considerable attention, the examination of pre-adoption and pre-implementation stages of configurable IT uptake appears largely under-investigated. In order to fulfill this research gap, a longitudinal case study was undertaken in which the periods prior the effective implementation of a CIS by two Canadian university multi-hospital centres were explored in-depth. This investigation appears therefore innovative at a methodological level because it adopts a process-based approach and a longitudinal design. In effect, whereas prior research exists on medical patient records implementation, most of these studies are factor-oriented, i.e., they seek to explain implementation in terms of relationships between dependent and independent variables. The work follows therefore the advice of the organizers of the Symposium 'Doing Longitudinal Studies of Health Care Change: Studying Health Care Change' at the 2010 Academy of Management Annual meeting: "In-depth longitudinal case studies of health care change are needed to better understand how these processes occur and how they might be more successfully managed" [48].

Our investigation makes two major theoretical contributions that, due to the exemplary quality of the cases involved, can constitute a useful tool to inform decision-making processes at the managerial level regarding early stages of the adoption of leading-edge IT in health care institutions elsewhere. The first is *to put forward the crucial role played by top managers' initial decisions and actions, discursively enacted, in the structuration of a configurable IT*. The lack of purposive strategies for involving clinicians from the very beginning of the project and for doing a preliminary diagnostic of existing clinical practices and the impact of the new system on these practices, can be understood as consequences of the path of dependency on external funding being built and accepted by all parties. If evidence of the critical components for CIS project success exists, how do the CIS top managers justify that their organizational decisions do not take them into account? The justifications, in our study, rely on external circumstances, meaning beyond their scope of responsibility. In these circumstances, top managers have displayed a set of discursive practices to convince everyone that they are doing right with the resources they have. They are aware of some of the likely consequences of their initial decisions, and of the fact that they are setting up a path of dependency vis-à-vis external agents. Nonetheless, they put forward a discourse indicating that everything that is under their control is being well managed and that they are able to change or alleviate adverse consequences that may emerge.

Furthermore, our investigation also contributes to the current trend of organizational studies that emphasizes the importance of rhetoric in organizational life. More particularly, we propose *rhetorical ambivalence *as a discursive device used by knowledgeable and reflective managers to monitor their behaviour and others' in order to initiate/sustain the adoption of a new IT in highly ambiguous institutional contexts. Rhetorical ambivalence thus enriches the existing repertoire of managerial discursive strategies --e.g. when a same person (a manager) adopts or abandons particular discursive practices according to context [49]-- constitutive of organizational reality. In this sense, and along with Whittle et al. [50], we also think that "the subtle, reflexive, and situationally competent use of discursive devices in rhetoric plays an important role in the process of organizational change". Indeed, our and others' future research should further inform us about the level of accomplishment of such discursive managerial strategies.

## Competing interests

The authors declare that they have no competing interests.

## Authors' contributions

Both authors conceived the study, organized the fieldwork, attended organizational meetings, and collected documentary material. CH performed the analysis, and drafted the article. MP contributed to the interpretation of results as well as paper editing. Both authors read and approved the final manuscript.

## Pre-publication history

The pre-publication history for this paper can be accessed here:

http://www.biomedcentral.com/1472-6947/11/42/prepub
